# Optimizing nutrition and oral health for caregivers—intervention protocol

**DOI:** 10.1186/s13063-021-05589-8

**Published:** 2021-09-15

**Authors:** Irma Nykänen, Tarja Välimäki, Liisa Suominen, Ursula Schwab

**Affiliations:** 1 Institute of Public Health and Clinical Nutrition, School on Medicine, University of Eastern Finland, P.O. Box 1627, FI-70211 Kuopio, Finland; 2 Department of Nursing Science, University of Eastern Finland, P.O.Box 1627, 70211 Kuopio, Finland; 3 Institute of Dentistry, School on Medicine, University of Eastern Finland, Kuopio, Finland; 4 Department of Oral and Maxillofacial Diseases, Kuopio University Hospital, Kuopio, Finland; 5 Institute of Clinical Medicine, Internal Medicine, Kuopio University Hospital, Kuopio, Finland

**Keywords:** Older people, Human, Diet, Nutrition, Oral health, Quality of life

## Abstract

**Background:**

The focus of care has shifted from institutional care to home care. Family caregivers provide the majority of home care that allows an opportunity for their care recipients to live at home and avoid costly institutional care. The aim of this study is to describe the nutritional status, oral health, and quality of life of family caregivers over the age of 65 and their care recipients, and to evaluate the impact of individually tailored diet and oral health advice to their nutritional status and oral health.

**Methods/design:**

Altogether, 250 family caregivers aged 65 or over, and their care recipients are studied in this prospective randomized population-based multidisciplinary 6-month intervention study. Participants are randomly allocated to the intervention groups or the control group. Data collection is performed at three time-points: at baseline and 6 months and after a 6-month follow-up at 12 months. Caregivers’ and their care recipients’ nutritional and oral health status as a primary outcome, and functional ability, cognitive status, quality of life, depression symptoms, sense of coherence, morbidity, and medication of family caregivers as secondary outcomes will be measured using validated self-administered questionnaires and clinical examinations.

**Discussion:**

To our knowledge, this is the first experiment to determine whether caregivers and their care recipients benefit from individual nutritional intervention and oral health intervention in terms of nutrition status, oral health status, and quality of life.

**Trial registration:**

ClinicalTrials.gov NCT04003493. Registered on June 28, 2019

## Background

Family caregivers (FCs) take the main responsibility of home care of older people. Family care is currently a common way to organize care of older people even when care recipients’ (CRs) cognitive and functional abilities are severely decreased. FCs are most often spouses in pensionable age with their underlying illnesses [1]. To support FCs, some countries have implemented various forms of formal support, including monetary support and supply of services. Such formal support provided by municipalities can include financial support for Fcs, services, respite care and counseling, and training. In Finland, home care is supported by care’s allowance that includes allowance fees, necessary services for care recipient, leave for FC (3 days per month), and informal care support services [2]. In Finland, 2018, over 47,500 FCs were granted care allowance, of them 65 years or older were 27,591 [3]. Typically, family care is the round-the-clock responsibility of CR covering all basic and instrumental needs. Intensive caregiving correlates with a greater caregiving burden [4] which may affect nutritional and adequate nutrition intake of FCs themselves and impaired capability to prepare nutritional meals for care recipient [5]. Besides, some FCs already suffer from depressive symptoms and psychological stress [6] and significantly lower quality of life at the start of home care [1].

If the nutrition and oral health statuses of family caregivers are not good, older people’s functional ability and general health can decline. This endangers living at home. Therefore, the nationwide goal of shifting the focus of services to outpatient care will not succeed. To achieve this goal, research is needed; for example, how to care for and improve oral health and nutrition for FCs and how these affect their wellbeing, performance, and quality of life. Caring for older people is considered the most economically and humanly sustainable alternative older people care as the number of older people increases, and social services are reduced. The Ministry of Social Affairs and Health in Finland has also stated that the well-being and rehabilitation of FCs should be developed, and for this purpose, a study on the current situation is needed.

There are few studies on the nutritional status of FCs and care recipients and their relationship to each other. In one study, only 37% of dementia FCs 18% of their care recipients had a normal nutritional status (Mini Nutritional Assessment MNA> 24) [7]. Tombini et al. found that only 4% of patients with Alzheimer’s disease and 35% of their caregivers had normal nutritional status (MNA> 24) [4]. A Finnish study has found that elderly male caregivers (52%), and their care recipients with Alzheimer’s disease are at a greater risk of malnutrition than female caregivers (40%) and their care recipients [8]. There are no studies on the effect of individual tailored nutritional counseling on caregivers’ nutritional status. However, individual nutritional counseling given to older people in home care has been found to have a positive effect on nutritional status [9]. Aims of this study are [1] to describe the nutritional status, nutrients intake, oral health and oral care, and quality of life of FCs and their CRs aged 65 years or older and [2] to evaluate the effect of individually tailored dietary and oral health counseling on the nutritional status, oral health, functional status, and quality of life among FCs and their CRs and [3] to develop an operating model to maintain good nutrition and oral health in FCs.

## Methods

### Study design

**Fig. 1:**
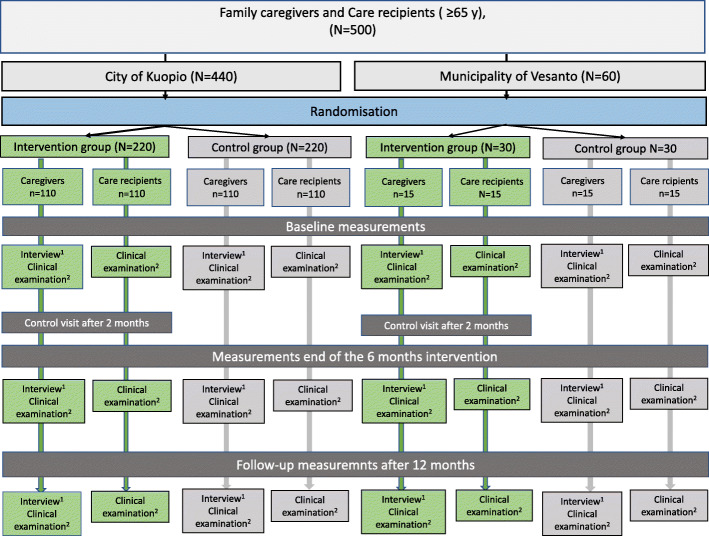
Flow chart of the study

This study is a prospective randomized population-based multidisciplinary intervention study. The study design is illustrated in Fig. 1. Participants are randomly allocated to the intervention group or the control group. Primary and secondary outcome measures are assessed at baseline and are repeated at the end of the 6-month intervention and 12 months after a 6-month follow-up. The trial was registered in the ClinicalTrials.gov Registry (NCT04003493), and the study plan was approved by the Research Ethics Committee of the Northern Savo Hospital District (Finland). The study is funded by a grant from the Sirkka and Jorma Turunen Foundation Finland. All participants are providing written informed consent before commencing the study. Participants may withdraw from participation at any time.

### Study population

Altogether, 250 FCs (Fig. 1) aged 65 years or over, and 250 CRs living in two areas in Eastern Finland will be recruited in this study. The researcher will record the participants, who will be willing to participate in the study and generate the allocation with the computer to assign participants to intervention. Due to the nature of the study, i.e., intervention on nutrition and oral health, blinding is not possible. So, participants and researchers are not blinded because it is impossible considering the design of the study. The FCs who have a caregiver’s allowance granted by municipalities valid on January 1, 2019, are included in the study.

The service managers for older people in Kuopio and Vesanto contact the caregivers first. They inform FCs about the study and ask them to indicate their willingness to participate in the study. Thereafter, the contact details of those interested are communicated to the researchers who are in contact with the volunteers by telephone. After that, they are receiving comprehensive oral and written information about the study and a consent form.

The sample size is based on power calculations on the main evaluation criteria of the intervention, the nutritional status (P-Alb), and the oral hygiene (plaque amount). In this study, power calculation showed that each subgroup required 60 subjects to achieve a statistical power of 0.80 with *α* of 0.05.

At the baseline, all the participants are interviewed and examined at home by a trained nurse, a clinical nutritionist, and a dental hygienist. Nutrition and oral health are measured at the baseline, at 6 months and 12 months. We have no exclusion criteria regarding maximum age, morbidity, or cognition. Subjects with end-of-life care are excluded. If a CR is unable to reply (e.g., in cases of severe cognitive impairment), data collection will be supplemented with an interview of her/his FC.

The control group does not receive the intervention, but they are guided to the health care, if their health status required it or they were at risk of malnutrition or if they need urgent dental care. If a participant is receiving concomitant treatment for, e.g., Alzheimer’s disease, Parkinson’s disease, diabetes, or cardiovascular disease, it will also be continued during the study.

The participants are encouraged to stay in the study from the beginning. In addition, all measurements are performed at home, giving the participants a better opportunity to participate in the study.

### Outcome variables

#### Interview and clinical examination by a clinical nutritionist

Nutritional screening is assessed with the MNA test [10]. The MNA is a widely used and validated tool for detecting malnutrition and its risk in older people. It involves a general assessment of health (questions regarding lifestyle, mobility, and drugs), a dietary assessment (questions regarding type and number of meals), anthropometric measurements, and a subjective self-assessment by the patient. The maximum sum score is 30.0; scores of 24.0–30.0 indicate normal nutritional status, scores of 17.0−23.5 a risk of malnutrition, and scores 0−16.5 malnutrition.

Bodyweight is measured to the nearest 0.1 kg by a beam scale with the subject wearing light clothes and without shoes. Height is measured standing, the head in the Frankfurt Plane position. If the participant is unable to stand, height is measured using indirect demi-span methods. Demi-span is the distance from the midline at the sternal notch to the web between the middle and ring fingers along outstretched arm. After that, height is calculated by a standard formula [11].

Concentrations of plasma albumin and prealbumin are measured according to standard protocols at the Eastern Finland Laboratory Centre ISLAB.

Grip strength of both hands is measured using a Saehan dynamometer (Hydraulic Hand Dynamometer, Saehan Corporation, South Korea) in a sitting position and with an elbow in 90° flexion close to the body. Participants are allowed two maximal efforts for both hands, and the mean value per hand is used.

#### Interview and clinical examination by a dental hygienist

A dental hygienist interviews the participants before carrying out a clinical oral health examination. The interview comprises of questions about the use and opinions of oral health services, self-reported oral health including the need for care, oral health-related quality of life, oral health-related behavior, attitudes towards oral health, and how caregivers assist care recipients in oral care

The dental hygienist conducts clinical examinations using mouth mirrors, WHO periodontal probes, and a headlamp with the participant sitting or lying down [12]. The subjects are first asked about the need for antibiotic prophylaxis in dental care, and if they answer yes, periodontal measurements are excluded. At first, the presence, type, and condition of removable dentures by the jaw are examined outside of the mouth. The need for repair is recorded as yes/no and denture hygiene will be marked as good if no visible plaque or calculus is found. Those wearing removable dentures are asked whether pain exits while using the dentures and if yes what kind of pain. The intraoral examination begins with an examination of the oral mucosa. After that, the number and condition (sound, filled, fractured, decayed, radix, or missing) of the teeth are recorded. The modified Silness and Löe index is used to register plaque [13]. Plaque is measured in the buccal surface of each tooth and registered as no visible plaque, visible plaque in gingival margins, visible plaque also elsewhere and the highest value per tooth is recorded. The number of mobile teeth is recorded after which the periodontal condition of the teeth is examined. Depths of the periodontal pockets are measured on every tooth except wisdom tooth and tooth remnants and registered as no deepened pocket, pocket 4–5 mm, or pocket ≥6 mm. Only the deepest pocket per tooth is recorded. The presence of gingival bleeding on probing is also registered from each tooth except wisdom teeth and recorded as yes, no, or unable to be examined/tooth missing. Possible needs and reasons for acute dental care are recorded, and further instructions to seek care are given to participants.

### Background variables

At the baseline, at 6 months and 12 months, trained nurse collects information about socioeconomic factors, living arrangements, general health status and health-related behavior, functional ability, cognitive status, quality of life, depression symptoms, sense of coherence, morbidity, and medication. Functional ability is assessed with the activities in daily living (ADL) index using the 10-item Barthel Index (scale 0–100), scores [14], 91–99 indicating slight dependency, 61–90 moderate dependency, 21–60 severe dependency and <20 total dependence, and instrumental activities of daily living (IADL) index using the 8-Item Lawton and Brody scale (from 0 to 8), higher scores indicating better functioning [15].

Cognitive status is assessed with the Mini-Mental State Examination (MMSE) on a scale from 0 to 30, scores 18–24 indicating mild cognitive impairment and scores <17 severe cognitive impairment [16]. The 15-item Geriatric Depression Scale (GDS-15) is used to assess depressive symptoms [17]. Quality of life is assessed with the World Health Organization Quality of Life (WHOQOL)–BREF [18] and sense of coherence (SOC) using Antonovsky’s [19] Orientation to Life Questionnaire (SOC-13). The SOC scale comprised of a general factor (SOC) with the three dimensions of comprehensibility (11 items), manageability (10 items), and 8 linesmeaningfulness (8 items).

Comorbidity is determined using a modified version of the Functional Comorbidity Index (FCI) [20]. The nurse records diagnoses (rheumatoid arthritis and other inflammatory connective tissue diseases, osteoporosis, diabetes, chronic asthma or chronic obstructive pulmonary disease (COPD), coronary artery disease, heart failure, myocardial infarction, stroke, depressive disorder, visual impairment, hearing impairment, Parkinson’s disease, multiple sclerosis, or obesity) based on primary care medical records. The investigator is determining the FCI index, where a higher FCI sum score indicates greater comorbidity.

The information on medication is collected using medication lists, packages, and prescriptions. We have used a cut-off of 10 drugs/day as excessive polypharmacy, according to Jyrkkä et al. [21].

All research methods in this study are summarized in Table 1.

**Table 1:** Study assessment and timetable

Assessment	Baseline assessments		2-month visit		6-month assessment		12-month assessment	
	FC	CR	FC	CR	FC	CR	FC	CR
Background information	x	x						
GHQ–12	x				x			
ADL	x				x			
IADL	x				x			
MNA	x	x			x	x	x	x
Albumin (g/dL)	x	x			x	x	x	x
Prealbumin (g/dL)	x	x			x	x	x	x
Weight, BMI	x	x			x	x	x	x
Grip strength	x	x			x	x		
SOC	x				x			
WHOQL-BREF	x				x		x	
GDS-15	x				x			
MMSE	x				x			
NPI	x				x			
FCI	x							
Oral health questionnaire^a^	x	x			x	x	x	x
Oral health clinical investigation^b^	x	x			x	x	x	x
Control visit^c^			x					

*FC* family caregiver, *CR* care recipient, *GHQ-12* General Health Guestionnaire-12, *ADL* activities of daily living, *IADL* instrumental activities of daily living, *MNA* Mini Nutritional Assessment, *SOC* sense of coherence, *WHO* Quality of Life Scale-Brief, *GDS-15* Geriatric Depression Scale-15, *MMSE* Mini-Mental State Examination, *NPI* neuropsychiatric inventory

^a^Oral health services, self-reported oral health, need for care, oral health-related quality of life, behavior, attitudes towards oral health, and how caregivers assist care recipients in oral care

^b^Removable dentures, denture hygiene, oral mucosa, number, and condition (sound, filled, fractured, decayed, radix, or missing) of the teeth, number of the mobile teeth, depths of the periodontal pockets, gingival bleeding, and plaque of the teeth

^c^Evaluation the implementation of nutrition and oral care plans and provide follow-up guidance as necessary

### Interventions

#### Nutritional intervention

The tailored nutritional intervention is based on the baseline MNA test and information on plasma albumin concentration and 4-day food records. Based on this evaluation, the nutritionist plans individualized nutritional care together with the FC and possibly also with the CR her/his nurse or relatives, if necessary. The nutritional care plan is developed for those who were at protein-energy malnutrition (PEM) or risk of PEM (MNA score <24 and plasma albumin <36 g/L. Those with insufficient energy or protein intake, or with weight loss, are instructed to increase their intake of energy nutrients by nutrient-dense food items and the number of meals and snacks. To increase energy intake participants are advised to eat more frequent small meals and snacks during the day and to increase the use of vegetable oils in foods and use high-fat vegetable oil-based spreads on bread. To increase protein intake, participants are advised to use dairy products like milk or sour milk as a drink, cheese on bread, and quark, yogurt, curdled milk, and cottage cheese as snacks and to eat daily meals with meat, poultry, fish, or sources of plant protein. The participants are given both daily meals and oral health care written instructions.

#### Oral health intervention

Based on the dental hygienist interview and the clinical examination, an individually targeted preventive intervention is carried out on those participants in need. The intervention includes at least one of the following verbal and written instructions: teeth brushing, interdental cleaning, cleaning and storing dentures, cleaning of the oral mucosa, dry mouth care, or some other. Intervention instructions are given to the FC.

At the 2-month visit, a nutritionist and a dental hygienist will evaluate the implementation of the nutrition and oral care plans and provide follow-up instructions as needed. Final measurements, the same as at the beginning of the study, will be made at the end of the intervention at 6 months. Follow-up measurements on nutrition and oral health will be made at 12 months. The intervention is terminated if a participant dies or is unable to continue the study due to worsening of his/her condition.

### Statistical analyses

Analyses will be performed using SPSS version 25.0 (IBM Corp. IBM SPSS Statistics for Windows, Armonk, NY). At baseline, statistical comparisons will be made between the groups using the chi-square test or *t* test (normally distributed variables) and Mann-Whitney’s *U* test (non-normally distributed outcomes). The effects of the intervention will be analyzed by generalized linear models, in which the baseline data will be included as covariates, the outcome measures as the dependent factor and group as the fixed factor. If necessary, both subgroup and custom analyses are used.

If the focus of the missing data is a small proportion of the observation units, they can be removed from the analysis. Prior to this procedure, it is checked whether the missing findings are randomly distributed among the or whether they focus on certain groups. In the latter case, removing the missing findings from the analysis may skew the results. The findings missing in the regression analysis can be removed in pairs. This means that when calculating the correlation matrix, all observation units that have information about two variables from which the correlation is calculated are considered. In this case, the data decreases, but not nearly as much as compared to the situation where all observation units containing missing data would be removed from the analysis.

## Discussion

In this study, FCs receive information to maintain and improve nutritional status and oral health and instructions on how to respond if their own or CRs’ nutritional status is poor or at risk of malnutrition or if they have oral hygiene problems.

The significance of family care to society and its economy must be recognized. Family care is considered the most sustainable alternative both economically and humanly sustainable alternative, because the number of elderly people increases. Helping and caring for a spouse is usually rewarding, but caring responsibilities can have significant consequences. FCs are usually older spouses with many diseases. This, combined with several years of continuous stressful family care, poses a significant risk to their own health and quality of life. This also impairs the health and quality of life of care recipients. Binding care can challenge the caregiver both physically and mentally, and narrow social relationships and other parts of life. Optimized nutrition combined with good oral health is expected to maintain the health, functional ability, and quality of life of both the FC and CR. Earlier studies have shown that good nutritional status accelerates recovery from illnesses and reduces the need for both short-term and long-term hospitalization, thus saving treatment costs.

The strengths of the present study are its population-based-controlled design and a wide variety of validated measurements with a multidisciplinary approach. Further, the strengths of this study are the diversity of the measures, including interviews and clinical examinations. The results of this study are directly applicable to real life. Also, the results will be used to develop an operating model to maintain good nutrition and oral health. This study has some limitations. The study is not a blinded study due to the study design. Some of the biases to which our research is susceptible are non-compliance with intervention guidelines and suspension during follow-up. In addition, the study population can only represent those FCs that were healthy enough to participate.

### Trial status

At the time of submission of the manuscript, this trial is in progress and participants are being recruited. The study began on January 1, 2019, and is expected to be completed by December 2021.

## Data Availability

Not applicable.

## Abbreviations

FCs: Family caregivers
CRs: Care recipients
MNA: Mini Nutritional Assessment
P-Alb: Plasma albumin
ISLAB: The Eastern Finland Laboratory Centre
WHO: World Health Organization
ADL: Activities of daily living
IADL: Instrumental activities of daily living
MMSE: Mini-Mental State Examination
GDS-15: The 15-item Geriatric Depression Scale
WHOQOL: World Health Organization Quality of Life
SOC: Orientation to Life Questionnaire
FCI: Functional Comorbidity Index
COPD: Obstructive pulmonary disease
PEM: Protein-energy malnutrition
